# A Study of Rough Set Approach in Gastroenterology

**DOI:** 10.1155/2013/782049

**Published:** 2013-11-27

**Authors:** Ahmet Sahiner, Tuba Yigit

**Affiliations:** Department of Mathematics, Suleyman Demirel University, 32260 Isparta, Turkey

## Abstract

We try to determine the type of abdominal pain of the patients who have several symptoms. Via the rough set theory, we obtain information table and discernibility matrix and put forward the status decision information. Thus, we obtain certain results and test these operations by the Rosetta program.

## 1. Introduction

Rough set theory was introduced by Pawlak in the early 1980s [1, 2]. The basic idea of this theory depends on classifying the objects that cannot be discernible according to some qualities. Rough sets can be defined using doubt, vagueness, and indeterminacy [3]. The theory can be used as a tool to discover data dependencies and to reduce the number of attributes contained in a data set requiring no additional information [4].

The most important feature of rough sets is that the theory is supported by mutual model development by practical exercise tools. In rough set, a large number of software systems are present. Rosetta and RSES can be given as an example. If we think of the problem of making groups of members which have a large number of qualifications in the set, the increasing number of members and qualities of members makes us insufficient to solve the problem.

Abdominal pain is one of the most common complaints that everybody may have at least once or a few times and one of the most important complaints that causes patient to go to doctor. Acute abdominal pain or acute abdominal as is called in surgery includes pathologies occurring with pain in abdominal region depending on the reasons except trauma which may require medicine or medical surgery. The reasons constituting clinical table include a lot of pathologies from mild to serious. Delays in diagnosis and cure may affect the success remarkably. Although there are many new and comprehensive methods by means of technological innovations, detailed story, careful inspection and doctor's predecisions are still too important.

Gastrointestinal infection (infectious intestinal disease) can be caused by a variety of communicable diseases and infections, which gain entry by and/or affect the gastrointestinal tract.

Infectious intestinal disease affects as many as 1 in 5 members of the population each year.

In this study data of the patients (who have abdominal pain) had been collected by the doctors who are employed in a private hospital at internal diseases clinic. The data of 58 patients suffering from these diseases have been examined. As the result of that examination the number of symptoms has been limited by 10. It is provided that the symptoms are diagnosed quickly by using minimum symptoms in the analysis part. Thus a leading data analysis has been done for decision periods of doctors.

## 2. The Concept of Rough Set

A data set is represented as a table, where each row represents a case, an event, a patient, or simply an object. Every column represents an attribute (a variable, an observation, a property, etc.) that can be measured for each object; the attribute may be also supplied by a human expert or user. This table is called an information system. More formally, that is a pair  *A* = (*U*, *A*), where  *U*  is a nonempty finite set of objects called the universe and  *A*  is a nonempty finite set of attributes such that  *a* : *U* → *V*_*a*_. The set is called the value set of  *a* · *a*(*x*)  denotes the value of attribute a for object  *x*. Any subset  *B*⊆  *A*  determines a binary relation  IND(*B*)  on  *U*, called indiscernibility relation, if  *a*(*x*) = *a*(*y*), for every  *a* ∈ *B*.

The family of all equivalence classes of  IND(*B*), namely, the partition determined by  *B*, will be denoted by  *U*/*B*. An equivalence class of  IND(*B*)  containing  *x*  will be denoted by  *B*(*x*). If  (*x*, *y*) ∈ IND(*B*)  we will say that  *x*  and  *y*  are  *B*-indiscernible.

Given an object subset  *X* ⊂ *U*, we call$\;\underline{B(x)}\;$and$\;\bar{B(X)}\;$the  *B*-lower and  *B*-upper approximation of  *X*, respectively;$\;\underline{B(x)}\;$and$\;\bar{B(X)}\;$are defined as follows:

(1) B(x)_={x∈U:B(x)⊆X},B(X)¯={x∈U:B(x)∩X≠∅}.

$\text{B}{\text{N}}_{B}\left(X\right)=\bar{B\left(X\right)}-\underline{B\left(x\right)}\;$is referred to as the  *B*-boundary region of  *X*. If  BN_*B*_(*X*) = *∅*, then the set  *X*  is exact with respect to  *B*, or  *X*  is referred to as rough set with respect to  *B*.

*R*  is a family of equivalence relations,  *r* ∈ *R*; if  IND(*R*) = IND(*R* − *r*), we say that  *r*  is indispensable in  *R*.

Given  *Q*⊆*R*,  IND(*Q*) = IND(*R*), and for any  *q* ∈ *Q*,  *q*  is indispensable. We say that  *Q*  is a reduct of  *R*. Obviously, there is not only a single reduct mostly. Core is defined as the common part of all reducts:

(2) Core=∩  red(R),

where  red(*R*)  denotes all of the reducts of  *R*. Reduct and core are two fundamental concepts of rough set theory. The reduct is the essential part of the information system, which can discern all objects discernible by the original one [5–13].

## 3. Data Set

### 3.1. List of Attributes of Symptoms

According to the information provided above, in order to be able to diagnose the related diseases distinctively we investigate answers to questions consisting of the following ten items. The letter  *a*  stands for attribute:

 (*a*_1_)  severity of abdominal pain ((1) mild, (2) medium, or (3) severe);
 (*a*_2_)  features of abdominal pain ((1) heavyset, (2) agonizing, or (3) reflecting pain);
 (*a*_3_)  characteristics of abdominal pain ((1) steady or (2) changeable);
 (*a*_4_)  duration of abdominal pain ((1) sudden start, (2) for days, (3) for months, or (4) for years);
 (*a*_5_)  location of abdominal pain ((1) right upper dial, (2) epigastric, (3) left upper dial, (4) umbilical, (5) right hypochondrium, or (6) left hypochondrium (7) pervasive);
 (*a*_6_)  relation to eating, ((1) increasing, (2) decreasing, or (3) steady);
 (*a*_7_)  the existence of alarm symptoms (anemia, losing weight, and rectal bleeding) ((1) alarm symptom exists, (2) alarm symptom does not exist);
 (*a*_8_)  the existence of systemic symptoms (fever, strong heartbeat, and low tension) ((1) systemic symptom exists (2) systemic symptom does not exist);
 (*a*_9_)  stomach intestine discomfiture ((1) bloated feeling-constipation, (2) bloated feeling-diarrhea, (3) bloated feeling-constipation-diarrhea or (4) no discomfiture);
 (*a*_10_)  the existence of dyspeptic complaints ((1) nausea-vomit, (2) burning-souring, (3) bitter liquid taste in mouth, or (4) pyrosis).

## 4. Clinical Table

The most significant symptom in the acute abdomen chart is the abdominal pain. Since the patient arrives at the clinic with a complaint of abdominal pain, the beginning of the history should also be with abdominal pain. The localization of pain and its beginning and development characteristics should be considered.

Different characters of abdominal pain in a patient with acute abdomen carry significant signs/traces/footprints in the diagnosis of the illness. It is possible to list those characters under the headings as follows:

1. reflection feature,
2. severity/intensity,
3. beginning and development feature,
4. form and period (frequency),
5. relation with food.

To give a brief summary of the illnesses examined in this context, we state the following.

*Cholecystitis (Inflammation of Gallbladder)*. It is an illness caused by the inflammation of the gallbladder. It usually develops depending on the stone in the gallbladder. It comes, suddenly or within hours, as agonizing pain localized in the upper right quadrant along with systemic symptoms such as fever, nausea, and vomiting.

*Peptic Ulcer*. It is an illness characterized by the increase of acid-pepsin secretion in the stomach. It is an inflammatory illness of the stomach. It is an illness whose most significant symptoms are heavyset burning-scraping pains whose reflections can sometimes be felt on shoulders and back. Stress and oily-spicy foods may trigger this illness.

*Irritable Intestinal Syndrome (Delicate Intestinal Illness)*. It is an illness especially of the large intestine. There has been an increase in the frequency of the illness recently. There is an increase in the sensitivity of the nerve cells in the large intestine mucosa. It does not have a sudden start. It is characterized by a pain in the abdominal region, swollenness, constipation, and/or diarrhea attacks.

*Pancreatic Inflammation*. It is an inflammatory illness of pancreas. It occurs suddenly; generally it may cause pervasive abdominal pain. One of the most common causes is long-term excessive alcohol consumption. Other causes include

- high levels of calcium in the blood;
- abnormalities in anatomy which are usually present at birth;
- cystic fibrosis;
- high blood fats (hypertriglyceridemia);
- in rare cases, some drugs can cause pancreatitis;
- in a number of cases no specific cause can be identified, a condition known as idiopathic pancreatitis.

*Reflux*. When you have something to eat or drink, it passes down the oesophagus (gullet) into the stomach. The flow of traffic should definitely be one way. However, reflux occurs when whatever happens to be in your stomach travels in the wrong direction back up into the oesophagus. Unlike vomiting, which is quite a violent activity, reflux mostly occurs without us being aware that it is happening.

Poor diet is believed to be the most prevalent acid reflux cause. Acid reflux occurs during digestion, when the stomach churns up acid or refluxes into the esophagus, causing a burning sensation in the chest or throat. Too much acid can push back through a valve between the stomach and the esophagus called the lower esophageal sphincter (LES). Along the same lines as diet, overeating also causes reflux. When you overeat, the stomach cannot keep up with the demand to process all the acids. So food gets backed up, and digestive acids infiltrate the esophageal valve to cause that unpleasant burning feeling centered in the chest.

Other factors that create a predisposition for acid reflux include smoking, use of alcohol, food allergies, certain medications, and lying down after meals.

The most frequent symptom is heartburn which is a burning sensation in the chest. Run heartburn is often most noticed at the lowest end of the bone, and the discomfort rises upwards to an extent that varies from individual to individual. Sometimes the burning feeling can reach all the way up to the throat. Heartburn occasionally can be felt deeply within the chest—almost within the back. Some patients notice reflux when some of the contents of their stomach “repeat” by coming back up the esophagus as far as the throat or even the mouth.

*Enteric*. It is an inflammation of intestine mucus and generally caused by bacteria and infections. It comes into existence sometimes abruptly and sometimes within hours as agonizing pains. Enteric symptoms included nausea, vomiting, abdominal pain, flatulence, tenesmus, fecal urgency, and incontinence.

It is an inflammation of intestine mucus and generally caused by bacteria and infections. It comes into existence sometimes abruptly, sometimes within hours as agonizing pains. Enteric symptoms included nausea, vomiting, abdominal pain, flatulence, tenesmus, fecal urgency, and incontinence.

## 5. Reduction and Status Decision

### 5.1. Collection of Data Bases

In this section, data of patients with abdominal pain provided through a questionnaire and converted into symptoms table attribute list"; hence we are ready to reduct the collected data by the technique of Rough Set theory. The list of attribute of symptoms consists of 10 basic attributes. In fact we have an information table including 58 different patients' symptoms. But for the sake of simplicity Table 1 is constructed according to only 29 different patients' symptoms.

The patients are shown as “*Y*”, and they are numerated as  *Y*_1_, *Y*_2_,…, *Y*_58_  in order. The attributes are shown as letter “*a*” and are numerated as  *a*_1_, *a*_2_,…, *a*_10_  in order. This  58 × 10  matrix is constructed according to attribute list obtained by complaint questionnaire. The numbers in the cells denote the value according to (3) as follows. Example of discernibility matrix for  (*Y*_1_, *Y*_2_, *Y*_58_)  is

(3) (  Y1Y7Y58Y1Y7a5,a6  a1,a2,a4,a1,a2,a4,Y58a5,a8,a9,a6,a8,a9,a10a10),

the table which was found as the result of making the data numerical. A column  *H*  showing the diagnosis of the patients will be added after that. The data set

(4) $$\begin{array}{c} H_{i}=\left\{\begin{array}{c} \text{IBS},i=1\\ \text{PU},i=2\\ \text{Enteritis},i=3\\ \text{Reflux},i=4\\ \text{Cholecystitiss},i=5\\ \text{Pancreatitis},i=6 \end{array}\right\} \end{array}$$

is arranged by column  *H*  in Table 1. The illnesses are numerated from 1 to 6 and are arranged in Table 1.

### 5.2. Discernibility Matrix and the Reductions

By the data taken from Table 1 our system becomes  58 × 58  symmetric discernibility matrix. The component  *c*_*ij*_  for  *i* ≠ *j*  of this matrix corresponds to symptoms attribute set. In this way, we have the opportunity to compare symptoms attributes of a given group of patients to those others, and then we can record these differences into the corresponding cell to obtain the discernibility matrix. The examples of rows and columns of this matrix for the cell  *Y*_1_, *Y*_7_, and  *Y*_58_  are as in (3).

Our real discernibility matrix will be filled by caring the symptoms differences among the patients in the disease groups in this way. This matrix is a diagonal symmetric. Using the discernibility matrix, the discernibility function can be found. The function  *f*_*A*_  is constructed as follows. Those are attributes within the same cell related by  (∨)  and those which are recorded in different cell are related by  (∧)  operation. Thus, our discernibility function is

(5) fA(a1,a2,a3,…,a58)=(a5∨a6,∨a10)∧(a1∨a3∨a4∨a5∨a7∨a10)∧(a1∨a5).

*f*_*A*_  discernibility function is simplified using the following features of the Boolean algebra:

(6) a∨(a∧b)=aa∧(b∧c)=aa∨(b∧c)=(a∨b)∧(a∨c)a∧(b∨c)=(a∧b)∨(a∧c).

When we have large data set, ROSETTA program can be used for analyzing the data tables. This program is used for data mining and knowledge processing. From given data bases, reduction process using several different algorithms reduces the mathematical overprocess. Language simplification process can be done in a short period by using “C++.” In this part reductions with different methods and status-decision knowledge were found and shown with the examples of output data.

### 5.3. Constitution of Reductions with Different Algorithms

In the following we give the resulting reductions obtained by using different algorithms. The data provided below are obtained using access data base linked by Rosetta program. By using this method only one attribution pair with 5 tuples. The other attributes are listed in 3 and 4 tuples has been found. See Tables 2, 3, 4, and 5. More precisely, for the sake of summarizing we can say the following.

Using Johnson method gives us the minimal reduction. It has been supported 100% by  {*a*_1_, *a*_3_, *a*_4_, *a*_5_, *a*_6_}  as can be seen in Table 7.

If we use RSES which is a directory library having been consisted of its own library of ROSETTA program and known to be consisted in using the experimental methods, we find the results in Table 4.

Table 4 looks like the table which is found by the method of genetic algorithm. The difference between genetic algorithm and RSES exhaustive is that latter one is limiting with 6 tuples term. In RSES genetic algorithm  *a*_3_  attribute appears as discernibility attribute of 6 different abdominal pain diseases. At this point, if we remove  *a*_3_  and  *a*_6_  dominant attributes and rearrange the remaining eight attributes we obtain the following order:

(7) a4→a3,  a5→a4,  a7→a5,a8→a6,  a9→a7,  a10→a8.

Then if we further apply reductions using the Rosetta program we obtain the results in Tables 6 and 7.

Having used this method, 4 tuples only two quality pair were found.

Table 7 gives us the information about the reduction result with respect to Johnson algorithm.

The output result  {*a*_2_, *a*_4_, *a*_5_, *a*_8_}  supports 100%. “Length” shows how many terms the reduction has.

Equation (8) is decision related discernibility matrix

(8) (Y1Y7Y58Y1a4a2,a4,a8Y7a4a2,a4,a8Y58a2,a4,a8a2,a4,a8).

## 6. Decision Relative Discernibility Matrices and Decision Relative Discernibility Functions

Essentially by using attributes  (*a*_2_∧*a*_4_∧*a*_5_∧*a*_8_)∨(*a*_2_∧*a*_4_∧*a*_7_∧*a*_8_)  with four terms, decision relative discernibility matrices and decision relative discernibility functions are found. Then two different matrices will occur for attributes with 4 tuples.

Functions will be constituted from these matrices and will be simplified. Decision relative discernibility matrix is diagonal symmetric such as in discernibility matrix. Each column of this matrix is written as multiplication of addition of attributes. By this way, decision relative discernibility functions are found by writing it as multiplication of addition of attributes for each patient. This function for  (*a*_2_∧*a*_4_∧*a*_7_∧*a*_8_)  is obtained from  (*a*_2_∧*a*_4_∧*a*_5_∧*a*_8_)  decision relative discernibility matrix shown in Table 8.

We used a Matlab program to get functions from this matrix. Then the following result has been found by doing individual simplifications:

(9) f1(A)=(a4∨a8)∧(a4∨a8)∧⋯∧(a2∨a4∨a7∨a8)=a4∧a7∧a8f2(A)=a4∧a7⋮f58(A)=a4∧(a2∨a8).

Decision relative discernibility function for  (*a*_2_∧*a*_4_∧*a*_5_∧*a*_8_)  is shown with  *g*_*i*_(*A*),  *i* = 1,…, 58  as the following:

(10) g1(A)=a4∧a8⋮g58(A)=a2∧a5∧a8.

### 6.1. The Decision Rules

A decision table is made through decision relative discernibility functions which are related to the decision described above. In the decision table, rows represent patients, columns represent groups of attributes with 4 terms.

The decision table can be accepted as a decision set. Now, we explain how this table is constructed in our study. For example, the condition and the decision for  (*a*_2_, *a*_4_, *a*_7_, *a*_8_)  can be found through the followings

(11) a46a71a82⇒H1a47a71a82⇒H1a47a71⇒H1a21a44a71⇒H1,a21a74a82⇒H2a22a42a83⇒H2a23a42⇒H2a23a42a74a82⇒H2.

Also the condition and the decision for  (*a*_2_, *a*_4_, *a*_5_, *a*_8_)  can be found through the followings

(12) a46a82⇒H1a21a52a84⇒H1a21a44a51a84⇒H1a46a82⇒H1,a21a42a82⇒H2a22a42a83⇒H2a23a42a52⇒H2a42a51a82⇒H2.

Finally, the above can be explained; for instance, if abdominal pain located left hypochondrium and meteorism and constipation, dyspepsia, the disease of the patient may be IBS; that is,

(13) a46a71a82⇒H1,

and if abdominal pain is colic and located epigastrium, dyspepsia, the disease of the patient may be peptic ulcer; that is,

(14) a22a42a83⇒H2.

## 7. Conclusion

We realized that rough set theory is very useful in classifying and analyzing a data set which consists of many attributes. This method could be used in many areas of science such as medicine, biology, and pharmacology. For instance, in pharmacology, reduction of adverse effects of a drug is very important.

Abdominal pain taken into consideration in this study is a symptom which we come across a lot in society. Although there are many, the most striking attributes of this pain related to this case has been determined by this method and this helps doctors diagnose with a great accuracy in a short time.

On the other hand, of course it is possible to support our theoretical in principle study with some clinical tests. In this case it would be possible to form multivariate matrix and we could analyze it by using cluster analysis, principal components analysis, and neuron net classification.

Planned future works are as follows.

1. Certain modern global optimization techniques such as fuzzy logic and intuitionistic fuzzy relations can be applied to this kind of problems.
2. The least and the most effective symptoms will be determined by using some classic and modern optimization tecniques together to prevent wastage on health care costs.

## Figures and Tables

**Table 1:** The symptoms for 29 of 58 different patients.

	*a*_1_	*a*_2_	*a*_3_	*a*_4_	*a*_5_	*a*_6_	*a*_7_	*a*_8_	*a*_9_	*a*_10_	*H*
*Y*_1_	1	1	1	4	6	1	2	2	1	2	**1**
*Y*_2_	1	1	1	4	7	3	2	2	1	4	**1**
*Y*_3_	2	1	2	3	4	1	1	2	1	4	**1**
*Y*_4_	1	1	1	4	6	3	2	2	3	2	**1**
*Y*_5_	1	1	2	4	6	1	2	2	1	4	**1**
*Y*_6_	1	1	2	4	4	1	2	2	3	4	**1**
*Y*_7_	1	1	1	4	3	3	2	2	1	2	**1**
*Y*_8_	1	1	1	3	3	1	2	2	3	4	**1**
*Y*_9_	2	2	1	4	4	1	2	2	1	4	**1**
*Y*_10_	1	1	2	3	6	3	2	2	3	4	**1**
*Y*_11_	1	1	2	4	7	1	2	2	1	4	**1**
*Y*_12_	1	1	1	4	4	1	2	2	3	2	**1**
*Y*_13_	1	2	1	3	6	1	2	2	1	1	**1**
*Y*_14_	1	1	1	4	7	1	2	2	1	4	**1**
*Y*_15_	1	2	1	4	5	3	2	2	1	2	**1**
*Y*_16_	1	1	1	3	2	1	2	2	3	4	**1**
*Y*_17_	2	2	1	4	7	1	2	2	1	4	**1**
*Y*_18_	2	1	1	4	6	3	2	2	3	4	**1**
*Y*_19_	2	1	1	3	2	2	2	2	4	2	**2**
*Y*_20_	2	2	1	2	2	2	2	2	4	3	**2**
*Y*_21_	2	3	1	2	2	3	2	2	4	2	**2**
*Y*_22_	2	3	1	3	2	2	1	2	4	2	**2**
*Y*_23_	2	3	1	3	2	2	2	2	4	2	**2**
*Y*_24_	1	3	1	3	2	2	2	2	4	2	**2**
*Y*_25_	2	3	1	2	4	3	2	2	4	2	**2**
*Y*_26_	2	1	1	3	2	2	2	2	1	2	**2**
*Y*_27_	2	3	1	3	3	2	1	2	4	2	**2**
*Y*_28_	1	3	1	3	7	2	2	2	4	2	**2**
*Y*_29_	2	1	1	3	2	2	2	2	4	2	**2**

**Table 2:** Reduction result by using Johnson Algorithm.

	Reduct	Support	Length
1	{*a*_1_, *a*_4_, *a*_5_, *a*_6_}	100	5

**Table 3:** Reduction result by using Genetic Algorithm.

	Reduct	Support	Length
1	{*a*_1_, *a*_2_, *a*_3_, *a*_7_, *a*_10_}	100	5
2	{*a*_2_, *a*_3_, *a*_4_, *a*_5_, *a*_6_}	100	5
3	{*a*_2_, *a*_3_, *a*_5_, *a*_9_, *a*_10_}	100	5
4	{*a*_1_, *a*_3_, *a*_6_, *a*_9_, *a*_10_}	100	5
5	{*a*_1_, *a*_3_, *a*_4_, *a*_9_, *a*_10_}	100	5
6	{*a*_1_, *a*_3_, *a*_5_, *a*_6_, *a*_10_}	100	5
7	{*a*_1_, *a*_2_, *a*_3_, *a*_5_, *a*_6_}	100	5
8	{*a*_2_, *a*_3_, *a*_5_, *a*_6_, *a*_10_}	100	5
9	{*a*_1_, *a*_3_, *a*_6_, *a*_7_, *a*_10_}	100	5
10	{*a*_1_, *a*_3_, *a*_4_, *a*_5_, *a*_6_}	100	5
11	{*a*_2_, *a*_3_, *a*_6_, *a*_7_, *a*_8_, *a*_10_}	100	6
12	{*a*_2_, *a*_3_, *a*_4_, *a*_6_, *a*_9_, *a*_10_}	100	6
13	{*a*_2_, *a*_3_, *a*_4_, *a*_7_, *a*_8_, *a*_10_}	100	6
14	{*a*_2_, *a*_3_, *a*_4_, *a*_7_, *a*_9_, *a*_10_}	100	6
15	{*a*_2_, *a*_3_, *a*_6_, *a*_7_, *a*_9_, *a*_10_}	100	6
16	{*a*_2_, *a*_3_, *a*_4_, *a*_5_, *a*_7_, *a*_10_}	100	6

**Table 4:** Reduction result by using RSES Exhaustive Algorithm.

	Reduct	Support	Length
1	{*a*_1_, *a*_2_, *a*_3_, *a*_5_, *a*_6_}	1	5
2	{*a*_1_, *a*_3_, *a*_4_, *a*_5_, *a*_6_}	1	5
3	{*a*_1_, *a*_3_, *a*_5_, *a*_6_, *a*_10_}	1	5
4	{*a*_1_, *a*_2_, *a*_3_, *a*_7_, *a*_10_}	1	5
5	{*a*_1_, *a*_3_, *a*_4_, *a*_9_, *a*_10_}	1	5
6	{*a*_1_, *a*_3_, *a*_6_, *a*_7_, *a*_10_}	1	5
7	{*a*_1_, *a*_3_, *a*_6_, *a*_9_, *a*_10_}	1	5
8	{*a*_2_, *a*_3_, *a*_4_, *a*_5_, *a*_6_}	1	5
9	{*a*_2_, *a*_3_, *a*_4_, *a*_7_, *a*_10_}	1	5
10	{*a*_2_, *a*_3_, *a*_4_, *a*_6_, *a*_9_, *a*_10_}	1	6
11	{*a*_2_, *a*_3_, *a*_5_, *a*_6_, *a*_10_}	1	5
12	{*a*_2_, *a*_3_, *a*_5_, *a*_7_, *a*_10_}	1	5
13	{*a*_2_, *a*_3_, *a*_5_, *a*_9_, *a*_10_}	1	5
14	{*a*_2_, *a*_3_, *a*_6_, *a*_7_, *a*_8_, *a*_10_}	1	6
15	{*a*_2_, *a*_3_, *a*_6_, *a*_7_, *a*_9_, *a*_10_}	1	6

**Table 5:** Reduction result by using RSES genetic algorithm.

	Reduct	Support	Length
1	{*a*_2_, *a*_3_, *a*_4_, *a*_7_, *a*_10_}	1	5
2	{*a*_1_, *a*_3_, *a*_6_, *a*_7_, *a*_10_}	1	5
3	{*a*_2_, *a*_3_, *a*_4_, *a*_5_, *a*_6_}	1	5
4	{*a*_1_, *a*_3_, *a*_4_, *a*_5_, *a*_6_}	1	5
5	{*a*_1_, *a*_2_, *a*_3_, *a*_7_, *a*_10_}	1	5
6	{*a*_1_, *a*_2_, *a*_3_, *a*_5_, *a*_6_}	1	5
7	{*a*_1_, *a*_3_, *a*_6_, *a*_9_, *a*_10_}	1	5
8	{*a*_2_, *a*_3_, *a*_5_, *a*_6_, *a*_10_}	1	5
9	{*a*_2_, *a*_3_, *a*_5_, *a*_7_, *a*_10_}	1	5
10	{*a*_1_, *a*_3_, *a*_4_, *a*_9_, *a*_10_}	1	5
11	{*a*_1_, *a*_3_, *a*_5_, *a*_6_, *a*_10_}	1	5
12	{*a*_2_, *a*_3_, *a*_5_, *a*_9_, *a*_10_}	1	5
13	{*a*_2_, *a*_3_, *a*_6_, *a*_7_, *a*_8_, *a*_10_}	1	6
14	{*a*_2_, *a*_3_, *a*_4_, *a*_6_, *a*_9_, *a*_10_}	1	6
15	{*a*_2_, *a*_3_, *a*_6_, *a*_7_, *a*_9_, *a*_10_}	1	6

**Table 6:** Reduction result by using genetic algorithm.

	Reduct	Support	Length
1	{*a*_2_, *a*_4_, *a*_7_, *a*_8_}	100	4
2	{*a*_2_, *a*_4_, *a*_5_, *a*_8_}	100	4
3	{*a*_1_, *a*_3_, *a*_4_, *a*_7_, *a*_8_}	100	5

**Table 7:** Reduction result by using Johnson algorithm.

	Reduct	Support	Length
1	{*a*_2_, *a*_4_, *a*_5_, *a*_8_}	100	4

**Table 8:** Reduction result by using RSES exhaustive.

	*a*_2_	*a*_4_	*a*_7_	*a*_8_	*H*
*Y*_1_	∗	6	1	2	**1**
*Y*_2_	∗	7	1	∗	**1**
*Y*_3_	1	4	1	∗	**1**
*Y*_4_	∗	6	3	2	**1**
*Y*_5_	∗	6	1	4	**1**
*Y*_6_	∗	4	3	4	**1**
*Y*_7_	1	3	1	2	**1**
*Y*_8_	∗	3	3	4	**1**
*Y*_9_	2	4	1	4	**1**
*Y*_10_	1	∗	3	4	**1**
*Y*_11_	1	7	1	∗	**1**
*Y*_12_	1	4	3	2	**1**
*Y*_13_	2	6	1	1	**1**
*Y*_14_	∗	7	1	∗	**1**
*Y*_15_	2	5	1	2	**1**
*Y*_16_	∗	7	3	∗	**1**
*Y*_17_	2	7	1	4	**1**
*Y*_18_	∗	6	3	4	**1**
*Y*_19_	1	∗	4	2	**2**
*Y*_20_	2	2	∗	3	**2**
*Y*_21_	3	2	∗	∗	**2**
*Y*_22_	3	2	∗	∗	**2**
*Y*_23_	3	2	∗	∗	**2**
*Y*_24_	3	2	∗	∗	**2**
*Y*_25_	3	2	4	2	**2**
*Y*_26_	∗	2	1	2	**2**
*Y*_27_	3	2	4	2	**2**
*Y*_28_	3	2	4	2	**2**
*Y*_29_	1	∗	4	2	**2**
*Y*_30_	3	4	4	2	**2**
*Y*_31_	3	1	∗	2	**2**
*Y*_32_	2	∗	2	∗	**3**
*Y*_33_	1	7	2	1	**3**
*Y*_34_	3	∗	2	1	**3**
*Y*_35_	1	5	2	1	**3**
*Y*_36_	3	∗	2	1	**3**
*Y*_37_	2	∗	2	∗	**3**
*Y*_38_	2	4	4	1	**3**
*Y*_39_	1	∗	4	3	**4**
*Y*_40_	1	∗	4	3	**4**
*Y*_41_	1	∗	4	3	**4**
*Y*_42_	∗	∗	1	3	**4**
*Y*_43_	∗	∗	1	3	**4**
*Y*_44_	∗	∗	1	3	**4**
*Y*_45_	1	∗	4	3	**4**
*Y*_46_	1	∗	4	3	**4**
*Y*_47_	∗	∗	1	3	**4**
*Y*_48_	∗	∗	1	3	**4**
*Y*_49_	∗	1	4	4	**5**
*Y*_50_	∗	1	∗	1	**5**
*Y*_51_	∗	1	∗	1	**5**
*Y*_52_	∗	1	∗	1	**5**
*Y*_53_	∗	1	∗	1	**5**
*Y*_54_	∗	1	∗	1	**5**
*Y*_55_	2	1	4	1	**5**
*Y*_56_	2	3	∗	1	**6**
*Y*_57_	2	7	4	1	**6**
*Y*_58_	1	7	∗	1	**6**

## References

[1] Marek W, Pawlak Z (1981). *Rough Sets and Information Systems*.

[2] Pawlak Z (1982). Rough sets. *International Journal of Computer & Information Sciences*.

[3] Pawlak Z (1991). *Rough Sets: Theoretical Aspects of Reasoning About Data*.

[4] Jensen R, Shen Q, Tuson A (2005). Finding rough set reducts with SAT. *Lecture Notes in Computer Science*.

[5] Yu D, Hu Q, Bao W Combining multiple neural networks for classification based on rough set reduction.

[6] Kondrad E, Orlawska E, Pawlak Z (1981). *An Approximate Concept Learning*.

[7] Michalski RS (1980). Recognition as role-guided inductive interference. *IEEE Transactions on Pattern Analysis and Machine Intelligence*.

[8] Orlwaska JE (1982). *Logic of Vague Concepts, Application of Rough Sets*.

[9] Starzyk A, Nelson DE, Strutz K (1999). Reduct generation in information systems, in information systems. *Bulletin of the International Rough Set Society*.

[10] Svozil K (1987). Quantum field theory on fractal spacetime: a new regularisation method. *Journal of Physics A*.

[11] Telçeken S (2003). *Kaba Kümeler Teorisi Yardımı ile Büyük Veri Topluluklarının Analizi, Yüksek Lisans Tezi*.

[12] Zhang L, Malik S The quest for efficient boolean satisfability solvers.

[13] Zhao Y, Yao Y, Luo F (2007). Data analysis based on discernibility and indiscernibility. *Information Sciences*.

